# Difficulties in teaching Grade 3 learners with reading problems in full-service schools in South Africa

**DOI:** 10.4102/ajod.v11i0.906

**Published:** 2022-02-10

**Authors:** Thembi A. Phala, Anna Hugo

**Affiliations:** 1 Department of Early Childhood Education, College of Education, University of South Africa, Pretoria, South Africa; 2 Department of Language Education, Arts and Culture, College of Education, University of South Africa, Pretoria, South Africa

**Keywords:** full-service schools, Grade 3 teachers, learner support teachers, reading problems, Bronfenbrenner’s ecological systems theory

## Abstract

**Background:**

Many primary school learners in South Africa, including those in so-called full-service schools (FSSs), are reading below their grade level.

**Objectives:**

The authors of this article embarked on a study to find out what difficulties a group of Grade 3 teachers in full-service school faced to support their young custodians with reading problems.

**Methods:**

The research followed a qualitative approach using semi-structured interviews and classroom observation.

**Results:**

The data showed that the reading problems experienced by some Grade 3 learners can be attributed to the multiple levels of the education system. On the macro system as set out by Bronfenbrenner’s ecological systems theory where the government and provincial departments operate, the first issue was the national department’s progression policy which allows Grade 2 learners to progress to Grade 3 without the required reading competency. The second issue was a lack of enough readers and overcrowding in classrooms. Problems on the microsystem included aspects such as the language of learning and teaching; learners’ reading skills and attitudes towards reading; teachers’ lack of knowledge about diverse learning needs and parental support.

**Conclusion:**

It is envisaged that the study will contribute to an understanding of the teachers’ difficulties in teaching reading so that the Grade 3 learners’ reading problems especially those in FSSs could be dealt with through combined efforts of all the stakeholders in the education system.

## Introduction

Reading is one of the most significant language skills that learners have to master at school, and it is important that this should be performed in the Foundation Phase (Grade 1 – Grade 3). It is a skill that permits learners to understand the meaning of written and printed material and a means of communication and language acquisition, sharing ideas and information (Reutzel & Cooter 2010:25). For Mercer, Mercer and Pullen (2011:263), reading is the most important pointer of achievement in life and school. In school, learners need to read more independently and comprehend the printed material when given tasks in the various subjects that they have to learn, especially after their first three years at school. Failure to read fluently and with comprehension results in high numbers of learners dropping out of school and could delay the learners’ learning potential (Pretorius et al. 2016:13). Reading problems could result in challenges at school and could also lead to the learners becoming stigmatised in the classroom.

Based on various reading assessments that were conducted over a couple of years in South African primary schools, there are many primary school learners who fail to read fluently and with comprehension. In studies conducted, the Progress in International Reading Strategy (PIRLS) revealed, for example, that in 2011 in South Africa 61% of learners could not read or write at the appropriate age levels and in 2016, 78% of Grade 4 learners were unable to read for meaning in any language, which included their home languages (HLs), respectively (Howie et al. 2017:2). In another study, the Annual National Assessment (ANA) revealed in 2014 that the national average performance in HL for Grade 3 learners stands at 56% (Department of Basic Education [DBE] 2014:41).

The group of Grade 3 teachers, who acted as participants in the qualitative study discussed in this article, were teaching in so-called full-service schools (FSSs) (to be discussed later). It is envisaged that by describing and understanding the difficulties that the teachers face when remediating the reading problems of learners, the teachers with the help of the school management will be in a better position to support the learners to acquire reading proficiency. During interviews with a group of teachers and during class observation, data were collected and the analysis of this data revealed the seven main themes. In the themes, problems that the Grade 3 teachers experienced became clear. If these problems could be addressed, the learners could acquire the necessary reading skills to make a success at school so that they could eventually become successful members of their communities.

### Inclusive education in South Africa

Inclusive education (IE) is a phenomenon which is embraced worldwide because of its principles of social justice, equity and access to quality education. In South Africa, the policy for IE was introduced in 2001, and it is defined as a system that recognises that all children and young people can learn, and that they require support to do so (Department of Education 2001:6). Inclusive education is therefore aimed at providing quality education to all learners regardless of their barriers to learning. Barriers to learning, according to the DBE (2014:vii), are the difficulties that arise within the learners, learning sites and education systems that preclude access to learning and development. Difficulties that arise within the learners are referred to as intrinsic barriers such as impairments, whilst those that arise outside the learners are referred to as extrinsic barriers, which could arise as a result of circumstances at school and in the environment.

In the South African Education White Paper 6 (EWP6) three types of schools to accommodate and support diverse learning needs of learners, including learners who experience reading problems, are identified. The three types of schools are mainstream schools, special schools which also act as resource centres (SSRCs) and FSSs (Department of Education 2001:21–22).

### Mainstream schools in South Africa

Mainstream schools are also referred to as ordinary or regular schools. The majority of learners in South Africa attend mainstream schools. These schools are set to cater for learners who require low-level need of support such as psychosocial support, visual or auditory processing problems (Gauteng Department of Education 2011:15). In order to build and maintain IE in future, it was decided by policymakers that teachers in mainstream schools should also receive support from the school districts, FSS and special schools as resource centres where and when needed.

### Special schools in South Africa

Special schools continue to exist as before in South Africa. These schools are strengthened so that they also serve as resource centres for mainstream schools and FSSs. These schools make provision for learners who require intensive levels of support such as extreme disabilities or extensive needs such as muscular or neurological disorders, who are hard of hearing or deaf, have low visions or who are blind, severely cognitively impaired and have serious behavioural disorders (Gauteng Department of Education 2011:16).

It was realised that there were learners in the mainstream in South African schools who had barriers to learning, but that they were not accommodated in the mainstream schools and special schools. These learners are predominantly those learners who had language problems and reading problems and those who had problems with mathematics. These problems are not as a result of an intellectual impairment. With regard to a reading problem, it could be as a result of an intrinsic barrier to learning such as dyslexia or as a result of external factors such as the socio-economic circumstances of learners, family factors, lack of resources at home and at schools and teachers’ training. These learners often became stigmatised as they did not make academic progress at school and could not keep up with the rest of the class. In order to provide additional support for these learners, FSSs were introduced.

### Full-service schools in South Africa

Full-service schools are intended to provide a quality equitable education to all learners, regardless of their learning abilities (DBE 2010:7). One of the reasons why so-called FSSs were introduced into the South African school system was to provide support to learners with mild-to-moderate barriers to learning, including reading problems (DBE 2010:21). With regard to inclusive education in South Africa, reference is made to intrinsic and extrinsic barriers to learning. Intrinsic barriers refer to conditions within a learner and extrinsic barriers that are conditions outside a learner. Moderate level of support refers to the type of support that the teachers can offer to learners who experience mild cognitive, hearing and visual impairments and learning problems including reading problems in their classrooms with additional assistance received from the therapists, counsellors or learner support teachers (LSTs). The additional assistance of a therapist, counsellor or LST would only be sought if necessary to attend to a specific need of a learner. Learner support teachers are qualified teachers who have specific knowledge of remedial education, special needs education and IE (Gauteng Department of Education 2004:3). In addition, Mahlo (2013:16) saw these teachers as having specialised skills and competencies to adapt the curriculum to suit the diverse learning needs of learners and strengthen support in FSSs. The teachers in FSSs rely on the Department of Education and District offices for additional training and improvement of their knowledge to support their learners. This is especially true when teachers in the Foundation Phase have to support learners with reading problems.

Strengthening support in FSSs requires a more collaborative effort from all stakeholders in the education system. Collaboration needs to happen inside and outside the school where different structures are working together. In FSSs, the following three collaborative structures are identified, namely the school-based support team, district-based support team and inter-collaborative structure (DBE 2010:22–23). The first structure is found in the school, which relates to Bronfenbrenner’s micro system, whilst the last two structures are found outside the school, which relate to Bronfenbrenner’s meso and macro levels. Bronfenbrenner’s ecological systems theory is the frame of reference in this article and will be discussed in the research methodology section.

### Reading in Grade 3 with reference to reading problems

Grade 3 is the last grade of the Foundation Phase. During the last year in this phase, learners should acquire the necessary skills ‘to learn to read’ to be able to ‘read to learn’ in Grade 4 and beyond (Spaull 2017). In the study discussed in this article, the Grade 3 learners were taught in their HLs, which were Sepedi and isiZulu, and in one school they were taught in English. This is in line with the language policy of the DBE. When they teach reading, teachers in the Foundation Phase have to know the various reading methods and reading strategies. In South Africa, Grade 3 learners are expected to read at grade level and also achieve a high level of communicative competence for acquiring reading competencies (DBE 2011:7–9). However, it is evident from research such as the *Progress in International Literacy Study* conducted in 2008 and 2016 (Govender & Hugo 2020:12) that most Grade 3 learners, including those in FSSs, have not reached the expected level in reading and are left behind as a result.

Children grow up and live under different circumstances and each living environment influences their motivation and ability to learn to read. A child’s home, school and the community form part of his or her environment. The central environment for any person is the home because this is where a young learner’s development and cognitive growth start (Jennings, Caldwell & Lerner 2010:25). At home, the parents could take a leading role to assist the learners in learning to read. Many studies have indicated the role of parents as agents towards influencing the learners’ reading behaviour. According to Van Bergen et al. (2016:147), there is a close link between children’s reading abilities and attributes of the family environment such as the educational achievements of the parents, if the parents read and whether reading material is available at home. However, in his research, Ramphele (2009:11) found that factors, such as high levels of poverty and socioeconomic status, could prevent parents from fulfilling this task.

The aim of the study is to help teachers and other persons such as the LST, therapists and the management team involved in education in primary schools to understand the reasons why Grade 3 teachers in FSSs, and for that matter in mainstream schools, experience difficulties to teach reading to their young custodians who have reading problems and whose reading abilities were below the standard expected of Grade 3 learners. There are many reading problems such as auditory memory, reversion of letters such as band or m and w, the blending of sounds or understanding the meaning of words. The reasons are to be found in various systems in children’s lives.

## Research methodology

A qualitative research approach was used in this study, as it was aimed to gain an understanding of the difficulties from the teachers’ point of view that they face whilst supporting Grade 3 learners in FSSs who experience reading challenges. According to Denzin and Lincoln (2020:3), qualitative research explains and reveals what occurs in true-to-life situations such as a school and a classroom. Creswell (2014:4) added that qualitative research provides researchers with the opportunity to investigate the meaning that people attach to personal and social problems.

In order to structure the study, purposive sampling was used to select the participants, and we selected teachers who had experience in teaching reading to Grade 3 learners. Grade 3 class teachers (GR3CT) and LST in FSSs were purposively identified from three FSSs in one of the districts in Gauteng (one of the provinces in South Africa). All FSSs were from township (in South Africa, in a suburb where predominantly black people live) areas. The specific FSSs were selected because of their geographical setting and accessibility for us to collect data. It was also based on the district’s Chief Education Specialist’s recommendation that the schools were practicing IE and that they were amongst the first round of mainstream schools in the district that were transformed into FSSs. In total, 18 teachers who comprised 12 GR3CTs and 6 LSTs were involved in the actual research, and another Grade 3 class teacher was involved in a pilot study. All the participants in the study were females and well-qualified primary school teachers. Eight of them had primary school teacher’s diplomas. Two had senior primary diplomas, one had a secondary school diploma, two had a BA degree and a teacher’s diploma and four had Honours degrees. The participants had many years of teaching experience ranging from 2 to 8 years.

### Data collection

Semi-structured interviews and classroom observations were used to obtain data. Verification of information received during the interviews against the information gathered from the classroom observation was facilitated by using multiple sources. It also enhanced the credibility of the research findings (Bertram & Christiansen 2016:209).

The interview questions comprised open-ended questions that allowed the participants to express their personal opinions and perceptions of what they think the causes of reading problems in their Grade 3 classrooms might be (Creswell & Poth 2016:92). Firstly, a pilot study was performed to validate the questions. Secondly, we observed the participants in their natural classrooms over a period of 3 weeks with the intention to further explore what the causes of reading problems in the Grade 3 classroom might be. According to the authors, some of the main causes of reading problems are phonological awareness, auditory processing difficulties, visual memory difficulties and comprehension of the language (Nel, Nel & Hugo 2016:222–227). An observation schedule was developed, and it included the following key areas: classroom layout, learner teacher support material and classroom practices. Another important aspect that we considered during the observation process was the context of the FSSs to understand how the context may influence the teachers’ challenges. In this article, the authors report on the research that was conducted in 2019 and thus no other more recent data are available.

The research thus involved more than one method and departed from a pragmatic approach. Pragmatism is a workable approach as it gives researchers the opportunity to do research in areas that interest them and then they can choose suitable methods (Armitage 2007:3). We also considered Bronfenbrenner’s ecological systems theory, which acted as a frame of reference and helped us to understand the various systems involved when teachers in Grade 3 classrooms in FSSs have to teach their learners to read. Bronfenbrenner’s ecological systems theory can be used to explain the influence of the environment on a person’s and also on a young learner’s biological, psychological, social and cultural development. The theory analyses the various systems in the environment, which contribute to the forming of the lives and lifestyles of people (Cala & Soriano 2014:50). The theory refers to the influence of situations to be found on micro, meso, exo and macro levels in a person’s life. When using Bronfenbrenner’s theory, the interrelatedness of systems that are involved in an education situation including the teaching of reading in Grade 3 classrooms becomes clear.

### Data analysis

The raw data collected during the interviews and classroom observation were analysed inductively to identify categories and themes (Bertram & Christiansen 2016:117). To thematically analyse the data, we adopted the process suggested by Creswell (2002 as cited in Leedy & Ormrod 2005:150), which sees data analysis as a spiral, moving from a narrow perspective to a broad one at the end. We listened to the recorded responses of the participants gathered during the interviews and then transcribed the data. We then reviewed the transcribed data several times until we identified general categories, themes and subthemes. The data collected from the observation were also incorporated and triangulated with the data from the interviews.

### Ethical considerations

An ethical clearance letter was obtained from the UNISA College of Education Ethics Review Committee (No. 2017/05/17/30112508/17/MC) and permission letters were obtained from the Gauteng Department of Education and from the principal of the three identified FSSs. The participants were requested to give consent in writing, indicating that they agreed to be interviewed and observed. The parents signed a letter of consent to indicate that they allowed their children to participate in the study and the learners were also asked to give assent.

## Findings

The results presented seven specific themes. The first four themes – the language of learning and teaching (LoLT) of the schools, learners’ lack of basic reading skills and abilities, teachers’ lack of teaching and facilitating skills to enhance learners’ reading levels, learners’ attitudes towards reading and the lack of parental support – refer to the micro level of Bronfenbrenner’s ecological systems theory. Other themes arising from the research were FSS teachers’ insufficient knowledge to plan and teach learners with diverse learning needs to read, lack of sufficient reading resources and seats and tables and the progression policy of the BW.

The participants whose direct quotations are used in this article formed part of a doctoral study. All of them were females and they were professionally trained primary school teachers and learner support teachers with three to 22 years of teaching experience. GR3CT refers to Grade 3 class teacher and LST refers to learner support teacher.

### Theme 1: Language of learning and teaching

Nationally, learners who do not speak the LoLT of the school seem to be the major challenge for many teachers (NEEDU 2016). In Grade 3, learners are expected to be taught in their HL. In the study, eight participants argued that in most cases, the HL of the learners was different from the LoLT of the school. This led to many of the Grade 3 learners not mastering reading and experiencing reading challenges because they could not understand what they were reading. The following comments support the statement (Phala 2019:199, 200):

‘I think the main problem is the language of learning and teaching in this school where I am because learners here are doing English, which is not their mother tongue. So, these learners they don’t know how to read because this language is not the language that they are using at home. They don’t have the basis in this language.’ (LST6, female, primary school teacher)

‘Okay, maybe the children at home they speak Zulu, Shangaan (Tsonga) or they speak another language but at school, the mother or usually their parents force it that the children must do Sepedi in school. Whereby at home they don’t speak Sepedi, so this gives us a big problem because when the learner you talk to him, he doesn’t understand you because she doesn’t speak the language. The big problem is the parents force their children to do Sepedi Home Language, but at home, they speak another language.’ (GR3CT1, female, primary school teacher)

In addition, another participant (GR3CT4) stressed that what made it even more difficult was the sound system of the different languages. She emphasised that isiZulu is more difficult to learn than English and Sepedi. If the learner did not have the background, he or she would struggle to know how to read and pronounce words. She (in Phala 2019:200) commented that:

‘I think from the isiZulu side, the sounds are difficult unlike in English and Sepedi. The Zulu sounds are difficult because the clicks like they have five letters that form one sound. Sounds like “indwa” like if the learners have difficulty with two letter sounds like “hl, sh, ng” when you add three letters on top of the two-letter sound it becomes difficult.’ (GR3CT4, female, primary school teacher)

The possible explanation of the reading problems arising from the language used to teach in the classroom was also linked to a lack of phonemic awareness, which is important when learning to read and which could affect the learners’ reading abilities. Thirteen participants in the study agreed that there were many learners who did not have the knowledge and understanding of the sound system of the LoLT in the schools and the sounds represented by the letters made it difficult for them to read. For the participants, this challenge was also linked to the methodologies that the lower-grade teachers used to teach learners to read. The following excerpts provide evidence of the participants’ concerns (Phala 2019:201):

‘… in my class is that the learners don’t know the alphabets, they confuse some of the alphabets. They don’t know the types of phonics, they don’t pronounce phonics, three words together, the vowels together. They cannot pronounce when the vowels are close to each other, when the consonants are together, they are unable to pronounce that.’ (GR3CT2, female, primary school teacher)

‘[*A*]ccording to me … for Grade 3 learners is that educators from Grade 1 and Grade 2 they did not teach the learners the letter sounds. They taught them the alphabet so the learners they only know the alphabet and not the letter-sound, and when they come to Grade 3, it becomes a problem because you have to start with the letter-sound and not the letter, so learners become confused. They are starting to be confused because they cannot differentiate between letter-sound and the alphabet.’ (LST5, female, primary school teacher)

In support of what the participants highlighted, we observed the challenges with the letter sounds during the classroom observation process. For example, in school C where English is the LoLT, before one participant started with the reading lesson, she asked the learners to read the following words: dog, big umbrella, bunny, little and beautiful from the board and most learners struggled to read the words. In her attempt to support them, she requested them to sound the letters, which was even more difficult. The learners were not acquainted with using letter sounds well and thus some of them confused the letter sounds with the alphabet names such as ‘d-o-g’ as ‘dee-o-gee’ and ‘b-i-g’ as ‘bee-ai-gee’. As a result, when the participant asked them to say the sounds of those letters again, they were frustrated and showed no sign of trying again.

### Theme 2: Learners lacking competences in basic reading skills and abilities

A number of participants referred to the learners’ lack of competence in the basic reading skills and abilities, which should have been acquired in Grades 1 and 2 as another challenge. Seven participants indicated that most of the Grade 3 learners who experienced reading problems were unable to read at all. Six participants highlighted that some learners with reading problems were unable to differentiate between sounds. Other challenges that were observed, included the reversal of the letters of the alphabet and the inability to pronounce letters. The following quotations illustrate the reading problems, as indicated by the participants (Phala 2019:215):

‘The challenge that I have is that the learners who are struggling [*and they]* cannot even pronounce or spell the letters.’ (LST2, female, primary school teacher)

‘The challenges are those learners who cannot differentiate “b” and “d” and be thinking that it is the same thing because they confuse the alphabets [*alphabet letters*] and those learners who cannot say “a” how to pronounce [*it*].’ (GR3CT, female, primary school teacher)

‘Most of them they can’t read because they don’t know the alphabets [*alphabet letters*]. We have also those who cannot read. They cannot combine the letters to make a word. Some learners can’t see that this is a “b” they see it as “p”. I also think the learners who doesn’t [*do not*] understand the phonics.’ (GR3CT7, female, primary school teacher)

The researchers observed that two of the participants referred to ‘alphabets’ when they actually meant ‘letters of the alphabet’. From the quotations, it is evident that many learners were experiencing reading problems and that the members of the teaching staff were aware of some causes of the reading problems as they were able to identify learners who struggled with the reversal of letters and an inability to pronounce words.

### Theme 3: Learners’ attitudes towards reading and their willingness to be supported

The findings revealed that seven participants were willing to teach the learners who experienced reading problems to read; however, the learners’ attitudes towards reading and their willingness to be supported raised concern. A participant reported (Phala 2019:215):

‘From the learners’ side they belittle themselves they feel somehow like we don’t know anything here at school and then they feel like we are giving this teacher a problem then it is okay. If we can just repeat, why do we have to try because we are not able to do what is supposed to be done.’ (GR3CT2, female, primary school teacher)

Another participant pointed out (Phala 2019:216):

‘There are those learners who will never read, who will read no matter how, he or she will never say a word and again. Some when they read, they spell, say word for word.’ (GR3CT3, female, primary school teacher)

Another participant also indicated her frustration and added another opinion. She linked the challenges to the learners’ disability and commented as follows (Phala 2019:216):

‘Sometimes you have realised that a learner has a reading problem, but it seems as if he or she doesn’t want to be assisted, he or she can sometimes keep quiet and not be interested in the lesson and usually when they read, they encounter problems with phonic problems. You may find that they don’t even recognise the phonics even though you taught them and you repeated them. Now and then you found that maybe they were not interested or whatever or maybe they are having hearing problems or whatever.’ (GR3CT5, female, primary school teacher)

This participant provided many possible reasons for learners’ reading problems. It became clear that a lack of motivation including motivation from the side of parents is often the reason why children do not want to be supported. Teachers could thus play a key role to motivate young learners to improve their reading abilities. The question arises whether the class teachers and LST in the FSSs who formed part of the research tried to find the exact reason or reasons for a learner’s reading problems so that these problems could be dealt with.

### Theme 4: Lack of parental support and involvement

The basis for young learners’ ability to learn to read starts at home. As a result, the teachers need to work together with parents to improve the reading skills of their children. Bronfenbrenner’s ecological systems theory acknowledges the interrelatedness of different systems for support to be effective (Swart & Pettipher 2016:11). Taking this into account, it is imperative to involve parents who have to support their children when learning to read to improve the children’s reading skills. From the data collected, three participants viewed a lack of parental support as another cause of reading problems. Parents need to encourage their children to read and to develop the culture of reading at home through storytelling. The three participants stated that some of the parents did not help their children with reading at home and the parents also did not read to their children. In addition, some of the learners stayed with their grandmothers who could not read at all.

One of the participants who was a learning support teacher expressed her personal experience and related the cause of reading problems to a lack of exposure to printed material and to parents who did not read to their children. She commented that as far as she knew the learners do not get exposure to any form of printing materials such as books and magazines. They tend to watch television or they played games that did not require reading (Phala 2019:202).

Another participant commented (Phala 2019:127):

‘My challenge is that parents are not involved when we give learners reading material to go and read at home. They are not helping their children so that is the biggest challenge because if the learner can read here at school and even at home, I think things will be a bit different.’ (LST6, female, primary school teacher)

Another issue related to the parents and the homes where the learners came from was the fact that some participants did not know the background of their learners. The statement is affirmed by a participant who said that sometimes they did not have the necessary background information about learners. When parents are requested to come to the school, they tend not to come. This meant that these learners could not be supported as the parents who knew a child better did not come to the school (Phala 2019:217). This was enhanced by yet another participant who opined that they were failing some of the learners because the parents did not do their part (Phala 2019:217).

Parents and the school are the main role players at the micro level of a child’s life. There should, therefore, be a close relationship between the parents and the school. The lack of a close relationship between the schools and the parents in this study was quite apparent from the participants’ comments. Consequently, most of these learners were not motivated to read as they lacked role models in reading. This statement corresponds with the argument by Serpell, Barker and Sonnenschein (2005:4) that the parents’ own literacy habits will influence their children’s interest and motivation to read. Alam (2021) who opined that parents’ literacy facilitates the development and establishment of their children’s reading habits.

### Theme 5: Full-service school teachers’ insufficient knowledge to plan and teach learners with diverse learning needs to read

Full-service schools admit learners who require mild-to-moderate levels of support. This calls for teachers to be more knowledgeable on how to plan and teach learners with diverse learning needs in their classrooms. Even though some participants acknowledged that they had attended workshops planned by the district officials, they highlighted that their lack of formal training in how to teach reading was not addressed well and this created many challenges for them. Hence, there was a plea from the participants for more training, especially on how to deal with learners with diverse reading problems. One LST remarked (Phala 2019:170):

‘We need more training on how to support these learners because every year teachers refer learners with different reading barriers and sometimes we are confused of what to do. The district office must supply us with more workshops on [*a*] continuous basis.’ (LST5, female, primary school teacher)

This theme relates to the provincial Department of Education under whose supervision the various districts reside, and this theme thus also refers to the meso level of Bronfenbrenner’s theory.

### Theme 6: Lack of sufficient reading resources and seats and tables

This theme, as well as the next theme, Progression Policy of the DBE, relates to the macro level of Bronfenbrenner’s theory. According to National Education Evaluation and Development Unit (2012:43), the issue of lack of enough learning–teaching support material was raised as an area of concern in South Africa. This comment refers to the national system and therefore relates directly to Bronfenbrenner’s macro level. From the findings, this issue was also highlighted as a challenge in the research study. Two participants commented as follows (Phala 2019:330, 327):

‘[*W*]e make photocopies for stories and then we start reading to the learners and then later the learners will read after us until we realise whether the learner is still struggling or is improving.’ (LTS5, female, primary school teacher)

‘I photocopy small letters those small letters – small alphabets.’ (GR3CT2, female, primary school teacher)

During the observation, the researchers also observed that not all learners had a reader that caters for their reading needs. In many instances, the teachers relied on photocopied text to be read to the learners. How can a young child learn to read and handle a book without a book? The lack of readers in FSSs where young learners with mild learning problems, amongst which are reading problems, have to be assisted, needs to be dealt with at national and provincial levels.

It seems that overcrowding and a lack of enough chairs and tables for learners remains a problem in many schools in South Africa, and this also needs the attention of the various departments of education. This is especially important in FSSs where the classroom and teaching circumstances should be conducive to each learner to make progress at school.

In the study, two participants stressed the issue of overcrowding. One participant said (Phala 2019:219):

‘But now our classes are full like for now in Grade 1 we are having 50 learners and in remedial education, they say if you are having five children who are cognitively challenged they are maybe like ten learners because they count one child times two.’ (GR3CT1, female, primary school teacher)

It is not in line with the policies of the DBE to have 50 learners in a class. In South Africa, the maximum teacher–learner ratio is 1 teacher to 40 learners (Motshekga 2012:2). This is, however, not the norm in all public schools as there are schools where the teacher–learner ratio is less.

### Theme 7: Progression policy of the Department of Basic Education

Three participants expressed their concern regarding the progression policy of the DBE. According to the assessment policy of the DBE, learners are not supposed to repeat twice in a phase. If it happens that a learner is still struggling, he or she will progress to the next grade with support. Three participants felt that some learners progressed to Grade 3 without being competent in reading. One participant remarked (Phala 2019:202):

‘In Grade 1 and 2, the learner did not do well, and they push the learner to Grade 3. That learner is going to have a problem.’ (GR3CT1, female, primary school teacher)

According to the researchers, this could pose a serious problem to a learner’s progress at school, and it could have a snowball effect. It is very difficult for a Grade 3 teacher to teach one reader in her class who cannot read as it is not part of the national curriculum to teach beginners reading in Grade 3. This is, however, what is expected of Grade 3 teachers and LSTs in FSSs, but it remains a difficult task. More advanced reading skills have to be taught in Grade 3 to prepare the learners for Grade 4 and beyond.

## Discussion

The aim of this article was to understand the challenges experienced by teachers when teaching Grade 3 learners who experience reading problems. The understanding was gained by reflecting on Grade 3 teachers’ challenges and the possible causes of reading problems of Grade 3 learners in FSSs. The teachers and LSTs who participated in this study viewed the challenges as complex and systemic. The teachers’ insufficient knowledge of different reading strategies limits their chances of addressing reading challenges in schools, especially in FSSs where learners with diverse reading needs are to be taught. Based on this, some teachers end up using a ‘one-size-fits-all’ approach. This is how they taught reading traditionally, but when this approach is used in schools, and especially in FSS, the specific reading needs of all learners are not attended to.

The teachers’ attitude to teach reading is influenced by their attitude towards reading and reading methods. Without relevant reading resources, such as graded reading books, challenges are created for teaching reading in FSSs. Schools should provide learners with enough and a variety of reading materials, and the provision should be made for the various reading levels of learners. Where possible it could be considered to assist learners especially those with reading challenges with well-designed computer-based reading support. If reading support is not provided, it could have a negative influence of the teaching of reading.

Overcrowding also poses a huge challenge for teachers. If classes are overcrowded, the teachers are unable to attend to each child individually; hence, a one-size-fits-all approach is adopted by most teachers. This is especially unacceptable in FSSs where learners with moderate barriers such as reading problems should be supported. The progression policy is deemed to aggravate the situation. When learners are not competent and they are progressed to the next grade, the next teacher faces challenges to teach these learners.

The lack of parental involvement creates a challenge for the extension of knowledge between the school and the home. If learners are only taught to read at school and not supported with their reading at home, it creates a gap, and this, in turn, reduces the learners’ chances to learn to read, using different environments and contexts.

When taking a closer look at the participants’ responses, we conclude that teachers’ challenges are complex and thought provoking and could be explained from different contexts or systems. The different contexts include the home, the school and the government departments. The interrelatedness of the various systems in the education structure shows how different factors from different contexts are closely connected and could contribute towards the reading ability or inability of learners to make progress at school.

The interrelatedness is evident when one considers Bronfenbrenner’s theory. This theory states that the way in which a child develops is influenced by the collaboration of the various systems in which he or she lives (Donald, Lazarus & Lolwana 2006:41–42). Within the context of this study, the factors in Bronfenbrenner’s micro system could be aligned as follows: LoLT; learners lacking competences in the basic reading skills; learners’ attitudes towards reading and their willingness to be supported; teachers’ insufficient knowledge to plan and teach diverse learners to read in FSSs and the lack of parental support and involvement. The teachers’ and LSTs’ need to be trained more to teach reading by the various district offices relates to the meso level of Bronfenbrenner’s theory. When Grade 2 learners are progressed to Grade 3 and they do not have the required reading competency, this is an issue at Bronfenbrenner’s macro system where the government operates. The issue of a lack of enough readers and overcrowding is also related to the macro system.

## Conclusion

The causes of the reading problems discussed in this article are to be found on more than one level of Bronfenbrenner’s systems theory. It became evident from the research that the classroom teachers and the LST are not the only ones to be blamed for the reading problems that a group of learners in FSSs experienced but that the reasons were to be found at many levels in the education structure.

It is important that the reading problems and the difficulties that Grade 3 teachers face to teach reading should be seen as part of a big system, and it requires that all subsystems work together to deal with the problems and difficulties. It is evident that the reasons why learners in the FSSs, which formed part of this research project, have reading problems call for an investigation of all the systems involved in the education structure.

We therefore conclude that the causes for reading challenges are systemic and that all the different stakeholders in the various systems involved in the teaching–learning situation in schools should be involved when attending to such challenges. It is hoped that Grade 3 learners with reading problems would not be stigmatised and left behind but that they would be supported by their teachers and other staff members to overcome their reading problems so that they could make a success of the years that they spend at school.
